# (2*S*,4*R*)-4-Fluoro­pyrrolidinium-2-carboxyl­ate

**DOI:** 10.1107/S1600536812031741

**Published:** 2012-07-18

**Authors:** David B. Hobart Jr, Joseph S. Merola

**Affiliations:** a Department of Chemistry, Virginia Tech, Blacksburg, VA 24061, USA

## Abstract

The crystal structure of the title compound, C_5_H_8_FNO_2_, at 100 K, displays inter­molecular N—H⋯O hydrogen bonding between the ammonium and carboxyl­ate groups as a result of its zwitterionic nature in the solid state. The five-membered ring adopts an envelope conformation with the C atom at the 3-position as the flap. The compound is of inter­est with respect to the synthesis and structural properties of synthetic collagens. The absolute structure was determined by comparison with the commercially available material.

## Related literature

For the synthesis of the title compound, see: Gottlieb *et al.* (1965 ▶); Azad *et al.* (2012 ▶). For its applications and properties with respect to synthetic collagens, see: Hodges & Raines (2003 ▶, 2005 ▶); Holmgren *et al.* (1999 ▶); Kim *et al.* (2005 ▶); Mooney *et al.* (2002 ▶); Persikov *et al.* (2003 ▶); Raines (2005 ▶); Shoulders & Raines (2009 ▶); Shoulders *et al.* (2006 ▶); Takeuchi & Prockop (1969 ▶). 
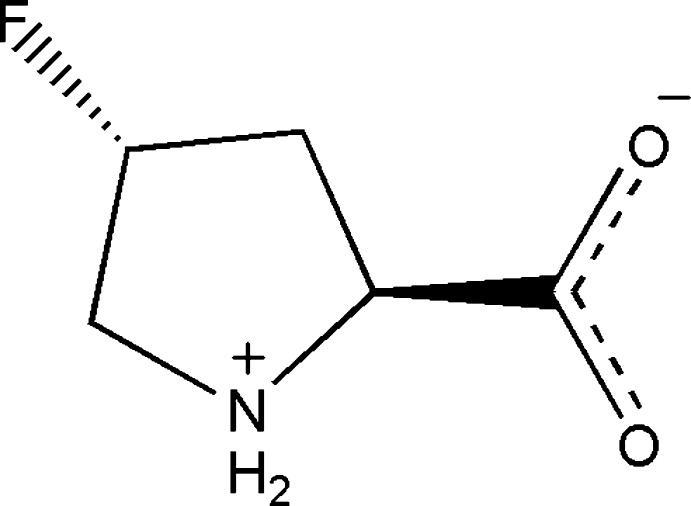


## Experimental

### 

#### Crystal data

C_5_H_8_FNO_2_
*M* *_r_* = 133.12
Orthorhombic, 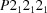

*a* = 7.6530 (6) Å
*b* = 8.4128 (6) Å
*c* = 8.6286 (6) Å
*V* = 555.54 (7) Å^3^
*Z* = 4
Mo *K*α radiation
μ = 0.14 mm^−1^
*T* = 100 K
0.26 × 0.05 × 0.03 mm

#### Data collection

Oxford Diffraction Gemini Ultra diffractometer
Absorption correction: Gaussian (*CrysAlis PRO*; Agilent, 2011 ▶) *T*_min_ = 0.977, *T*_max_ = 0.996
10227 measured reflections
959 independent reflections
832 reflections with *I* > 2σ(*I*)
*R*_int_ = 0.082

#### Refinement

*R*[*F* ^2^ > 2σ(*F* ^2^)] = 0.038
*wR*(*F* ^2^) = 0.073
*S* = 1.07
959 reflections
114 parameters
All H-atom parameters refined
Δρ_max_ = 0.32 e Å^−3^
Δρ_min_ = −0.23 e Å^−3^

### 

Data collection: *CrysAlis PRO* (Agilent, 2011 ▶); cell refinement: *CrysAlis PRO*; data reduction: *CrysAlis PRO*; program(s) used to solve structure: *SHELXS97* (Sheldrick, 2008 ▶); program(s) used to refine structure: *SHELXL97* (Sheldrick, 2008 ▶); molecular graphics: *OLEX2* (Dolomanov *et al.*, 2009 ▶); software used to prepare material for publication: *OLEX2*.

## Supplementary Material

Crystal structure: contains datablock(s) I, global. DOI: 10.1107/S1600536812031741/im2392sup1.cif

Structure factors: contains datablock(s) I. DOI: 10.1107/S1600536812031741/im2392Isup2.hkl

Supplementary material file. DOI: 10.1107/S1600536812031741/im2392Isup3.cdx

Supplementary material file. DOI: 10.1107/S1600536812031741/im2392Isup4.cml

Additional supplementary materials:  crystallographic information; 3D view; checkCIF report

**Fig. 1.**
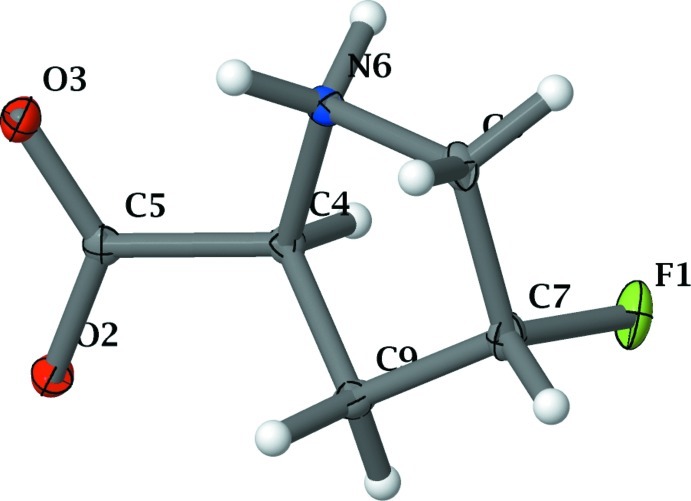
View of the title compound showing displacement ellipsoids at the 50% probability level.

Enhanced figure: interactive version of Fig. 1

## Figures and Tables

**Table 1:** Hydrogen-bond geometry (Å, °)

*D*—H⋯*A*	*D*—H	H⋯*A*	*D*⋯*A*	*D*—H⋯*A*
N6—H6*B*⋯O2^i^	0.92 (3)	1.90 (3)	2.744 (2)	152 (2)
N6—H6*A*⋯O2^ii^	0.91 (3)	2.01 (3)	2.899 (2)	164 (2)

Symmetry codes: (i) 
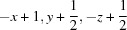
; (ii) 
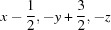
.

**Fig. 2.**
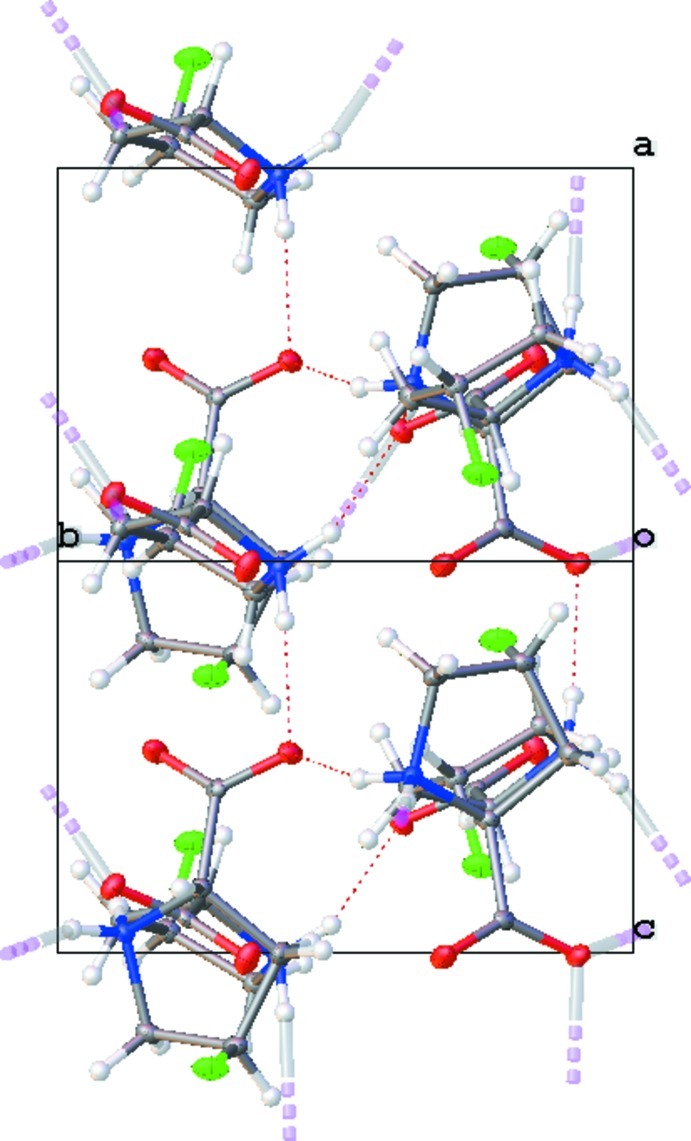
A view of a section of the crystal packing of the title compound along [101] showing N6–H6B···O2i and N6–H6A···O2ii hydrogen bonds [Symmetry code (i) 3/2 - x, 1 - y, 1/2 + z; (ii) +x, +y, 1 + z].

## supplementary crystallographic information

### Comment

The title compound is a useful building block in the synthesis of synthetic collagens. Collagen is the most abundant protein found in animals and exists as a triple helix comprised of three strands. The amino acid encoding of the strands follows the *X*—Y-Gly pattern. Trans-4-fluoroproline has been shown to induce hyperstability of the triple helix when substituted for the Y codon.

### Experimental

The title compound was purchased commercially from Bachem Americas, Inc., 3132 Kashiwa Street, Torrance, CA 90505 USA. Single crystals suitable for diffraction were grown *via* slow evaporation from a 50/50 (*v*/*v*) solution of acetone and water.

### Refinement

No Flack parameter is reported as Friedel pairs were merged *via* MERG3 instruction due to the absence of anomalous dispersion effects. Data collection was with Mo radiation and no heavy atoms are present. Chirality at each stereocenter was confirmed by comparison to the known stereochemistry of the commercially available material.

Hydrogen atoms were located from Fourier maps (Q-peaks) and all hydrogen atom parameters were refined.

### Crystal data

**Table:**

C_5_H_8_FNO_2_	*D*_x_ = 1.592 Mg m^−^^3^
*M**_r_* = 133.12	Mo *K*α radiation, λ = 0.71073 Å
Orthorhombic, *P*2_1_2_1_2_1_	Cell parameters from 1887 reflections
*a* = 7.6530 (6) Å	θ = 3.6–30.0°
*b* = 8.4128 (6) Å	µ = 0.14 mm^−^^1^
*c* = 8.6286 (6) Å	*T* = 100 K
*V* = 555.54 (7) Å^3^	Prism, clear light colourless
*Z* = 4	0.26 × 0.05 × 0.03 mm
*F*(000) = 280	

### Data collection

**Table:**

Oxford Diffraction Gemini Ultra diffractometer	959 independent reflections
Radiation source: fine-focus sealed tube, fine-focus sealed tube	832 reflections with *I* > 2σ(*I*)
Graphite monochromator	*R*_int_ = 0.082
Detector resolution: 16.0122 pixels mm^-1^	θ_max_ = 30.1°, θ_min_ = 3.6°
phi and ω scans	*h* = −10→10
Absorption correction: gaussian (*CrysAlis PRO*; Agilent, 2011)	*k* = −11→11
*T*_min_ = 0.977, *T*_max_ = 0.996	*l* = −12→12
10227 measured reflections	

### Refinement

**Table:**

Refinement on *F*^2^	Primary atom site location: structure-invariant direct methods
Least-squares matrix: full	Secondary atom site location: difference Fourier map
*R*[*F*^2^ > 2σ(*F*^2^)] = 0.038	Hydrogen site location: inferred from neighbouring sites
*wR*(*F*^2^) = 0.073	All H-atom parameters refined
*S* = 1.07	*w* = 1/[σ^2^(*F*_o_^2^) + (0.0203*P*)^2^ + 0.2288*P*] where *P* = (*F*_o_^2^ + 2*F*_c_^2^)/3
959 reflections	(Δ/σ)_max_ < 0.001
114 parameters	Δρ_max_ = 0.32 e Å^−^^3^
0 restraints	Δρ_min_ = −0.23 e Å^−^^3^

### Special details

**Table:**

Experimental. Recrystallized from 50/50 acetone/water.Absorption correction: CrysAlisPro, Agilent Technologies, Version 1.171.34.49 (release 20-01-2011 CrysAlis171 .NET) (compiled Jan 20 2011,15:58:25) Numerical absorption correction based on gaussian integration over a multifaceted crystal model
Geometry. All e.s.d.'s (except the e.s.d. in the dihedral angle between two l.s. planes) are estimated using the full covariance matrix. The cell e.s.d.'s are taken into account individually in the estimation of e.s.d.'s in distances, angles and torsion angles; correlations between e.s.d.'s in cell parameters are only used when they are defined by crystal symmetry. An approximate (isotropic) treatment of cell e.s.d.'s is used for estimating e.s.d.'s involving l.s. planes.
Refinement. Refinement of *F*^2^ against ALL reflections. The weighted *R*-factor *wR* and goodness of fit *S* are based on *F*^2^, conventional *R*-factors *R* are based on *F*, with *F* set to zero for negative *F*^2^. The threshold expression of *F*^2^ > σ(*F*^2^) is used only for calculating *R*-factors(gt) *etc*. and is not relevant to the choice of reflections for refinement. *R*-factors based on *F*^2^ are statistically about twice as large as those based on *F*, and *R*- factors based on ALL data will be even larger.

### Fractional atomic coordinates and isotropic or equivalent isotropic displacement parameters (Å^2^)

**Table:**

	*x*	*y*	*z*	*U*_iso_*/*U*_eq_	
F1	0.24111 (16)	0.73474 (16)	0.53792 (14)	0.0194 (3)	
O2	0.58809 (19)	0.59578 (17)	0.07960 (17)	0.0143 (3)	
O3	0.4982 (2)	0.82996 (18)	−0.01402 (17)	0.0177 (3)	
N6	0.2662 (2)	0.8837 (2)	0.2162 (2)	0.0109 (3)	
C4	0.3974 (3)	0.7510 (2)	0.2345 (2)	0.0104 (4)	
C5	0.5019 (3)	0.7239 (2)	0.0854 (2)	0.0104 (4)	
C7	0.1571 (3)	0.6913 (3)	0.3970 (2)	0.0130 (4)	
C8	0.1078 (3)	0.8427 (3)	0.3133 (2)	0.0124 (4)	
C9	0.2903 (3)	0.6117 (3)	0.2947 (2)	0.0137 (4)	
H4	0.480 (3)	0.787 (3)	0.317 (3)	0.010 (6)*	
H8A	0.011 (3)	0.822 (3)	0.247 (3)	0.013 (6)*	
H7	0.056 (3)	0.629 (3)	0.424 (3)	0.011 (6)*	
H8B	0.084 (3)	0.927 (3)	0.387 (3)	0.008 (6)*	
H9A	0.233 (3)	0.560 (3)	0.208 (3)	0.020 (7)*	
H9B	0.358 (3)	0.540 (3)	0.353 (3)	0.022 (7)*	
H6A	0.231 (3)	0.895 (3)	0.115 (3)	0.026 (7)*	
H6B	0.314 (3)	0.975 (3)	0.256 (3)	0.020 (7)*	

### Atomic displacement parameters (Å^2^)

**Table:**

	*U*^11^	*U*^22^	*U*^33^	*U*^12^	*U*^13^	*U*^23^
F1	0.0170 (6)	0.0320 (8)	0.0092 (6)	0.0011 (6)	0.0006 (5)	0.0004 (5)
O2	0.0151 (7)	0.0142 (7)	0.0136 (7)	0.0048 (6)	0.0027 (6)	0.0023 (6)
O3	0.0248 (8)	0.0158 (7)	0.0124 (7)	0.0051 (7)	0.0053 (7)	0.0031 (6)
N6	0.0115 (8)	0.0101 (8)	0.0112 (8)	0.0008 (7)	0.0000 (7)	−0.0014 (7)
C4	0.0110 (8)	0.0110 (9)	0.0091 (8)	0.0004 (8)	−0.0003 (7)	−0.0002 (8)
C5	0.0094 (8)	0.0113 (9)	0.0106 (8)	−0.0034 (8)	0.0000 (7)	−0.0015 (8)
C7	0.0119 (9)	0.0158 (10)	0.0114 (9)	−0.0018 (8)	0.0018 (8)	−0.0003 (8)
C8	0.0106 (9)	0.0153 (9)	0.0112 (9)	0.0017 (8)	0.0007 (8)	−0.0022 (8)
C9	0.0149 (10)	0.0118 (9)	0.0144 (10)	0.0009 (8)	0.0013 (8)	0.0035 (9)

### Geometric parameters (Å, º)

**Table:**

F1—C7	1.423 (2)	C4—H4	1.00 (2)
O2—C5	1.265 (2)	C7—C8	1.512 (3)
O3—C5	1.238 (2)	C7—C9	1.505 (3)
N6—C4	1.509 (3)	C7—H7	0.96 (2)
N6—C8	1.513 (3)	C8—H8A	0.95 (3)
N6—H6A	0.91 (3)	C8—H8B	0.97 (2)
N6—H6B	0.92 (3)	C9—H9A	0.97 (3)
C4—C5	1.532 (3)	C9—H9B	0.94 (3)
C4—C9	1.521 (3)		
			
C4—N6—C8	107.85 (15)	F1—C7—H7	107.3 (14)
C4—N6—H6A	111.6 (17)	C8—C7—H7	111.6 (13)
C4—N6—H6B	108.2 (15)	C9—C7—C8	105.27 (17)
C8—N6—H6A	108.5 (17)	C9—C7—H7	116.6 (14)
C8—N6—H6B	107.5 (15)	N6—C8—H8A	109.4 (15)
H6A—N6—H6B	113 (2)	N6—C8—H8B	110.1 (13)
N6—C4—C5	111.70 (16)	C7—C8—N6	104.86 (16)
N6—C4—C9	104.28 (16)	C7—C8—H8A	109.3 (15)
N6—C4—H4	105.8 (13)	C7—C8—H8B	110.5 (13)
C5—C4—H4	108.2 (13)	H8A—C8—H8B	112.4 (19)
C9—C4—C5	116.93 (17)	C4—C9—H9A	108.9 (15)
C9—C4—H4	109.3 (13)	C4—C9—H9B	112.4 (15)
O2—C5—C4	115.64 (17)	C7—C9—C4	102.86 (17)
O3—C5—O2	126.79 (19)	C7—C9—H9A	110.3 (15)
O3—C5—C4	117.53 (18)	C7—C9—H9B	110.2 (15)
F1—C7—C8	107.72 (17)	H9A—C9—H9B	112 (2)
F1—C7—C9	108.03 (17)		

### Hydrogen-bond geometry (Å, º)

**Table:**

*D*—H···*A*	*D*—H	H···*A*	*D*···*A*	*D*—H···*A*
N6—H6*B*···O2^i^	0.92 (3)	1.90 (3)	2.744 (2)	152 (2)
N6—H6*A*···O2^ii^	0.91 (3)	2.01 (3)	2.899 (2)	164 (2)

Symmetry codes: (i) −*x*+1, *y*+1/2, −*z*+1/2; (ii) *x*−1/2, −*y*+3/2, −*z*.
